# Whole-exome sequencing and digital PCR identified a novel compound heterozygous mutation in the *NPHP1* gene in a case of Joubert syndrome and related disorders

**DOI:** 10.1186/s12881-017-0399-2

**Published:** 2017-03-27

**Authors:** Shingo Koyama, Hidenori Sato, Manabu Wada, Toru Kawanami, Mitsuru Emi, Takeo Kato

**Affiliations:** 1 Department of Neurology, Hematology, Metabolism, Endocrinology, and Diabetology, Yamagata University Faculty of Medicine, 2-2-2 Iida-nishi, Yamagata, 990-9585 Japan; 2 Thoracic Oncology Program, University of Hawaii, Cancer Center, 701 Ilalo Street, Honolulu, HI 96813 USA

**Keywords:** Case report, Joubert syndrome, NPHP1, Deletion, Hidden Markov model, Whole exome sequencing, Digital PCR

## Abstract

**Background:**

Joubert syndrome and related disorders (JSRD) is a clinically and genetically heterogeneous condition with autosomal recessive or X-linked inheritance, which share a distinctive neuroradiological hallmark, the so-called molar tooth sign. JSRD is classified into six clinical subtypes based on associated variable multiorgan involvement. To date, 21 causative genes have been identified in JSRD, which makes genetic diagnosis difficult.

**Case presentation:**

We report here a case of a 28-year-old Japanese woman diagnosed with JS with oculorenal defects with a novel compound heterozygous mutation (p.Ser219*/deletion) in the *NPHP1* gene. Whole-exome sequencing (WES) of the patient identified the novel nonsense mutation in an apparently homozygous state. However, it was absent in her mother and heterozygous in her father. A read depth-based copy number variation (CNV) detection algorithm using WES data of the family predicted a large heterozygous deletion mutation in the patient and her mother, which was validated by digital polymerase chain reaction, indicating that the patient was compound heterozygous for the paternal nonsense mutation and the maternal deletion mutation spanning the site of the single nucleotide change.

**Conclusion:**

It should be noted that analytical pipelines that focus purely on sequence information cannot distinguish homozygosity from hemizygosity because of its inability to detect large deletions. The ability to detect CNVs in addition to single nucleotide variants and small insertion/deletions makes WES an attractive diagnostic tool for genetically heterogeneous disorders.

**Electronic supplementary material:**

The online version of this article (doi:10.1186/s12881-017-0399-2) contains supplementary material, which is available to authorized users.

## Background

Joubert syndrome and related disorders (JSRD) (OMIM 213300) is a rare neurological disorder with autosomal recessive or X-linked inheritance, characterized by neurological symptoms including hypotonia, ataxia, global developmental delay, abnormal eye movements, and breathing dysregulation [1–3]. JSRD is also often associated with visceral involvements and is classified into six clinical subtypes [2]. The neuroradiological hallmark of JSRD is the so-called molar tooth sign, which reflects a complex cerebellar and brainstem malformation [4, 5]. To date, 21 genes have been reported to be responsible for JSRD [1, 6]. JSRD has clinical and genetic heterogeneity, which makes it difficult to identify the causative mutations in individual cases by Sanger sequencing alone [7]. Whole-exome sequencing (WES) is becoming widely adopted as an efficient strategy to identify disease-causing mutations in genetically heterogeneous diseases. In addition to single nucleotide variants (SNVs) and small insertion/deletions, structural changes such as copy number variations (CNVs) contribute to human diseases [8, 9]; however, the accurate detection of CNVs using WES data remains challenging. In this study, WES and a read depth-based CNV detection algorithm using WES data of the family identified not only a novel nonsense mutation but also a large heterozygous deletion mutation in *NPHP1*, leading to the precise molecular diagnosis of JSRD.

## Case presentation

### Clinical report

**Fig. 1:**
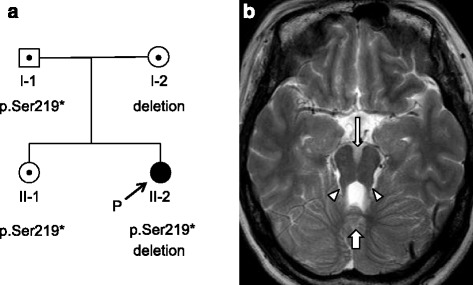
**a** Pedigree of the family. *Squares*: males; *circles*: females. The filled circle represents the patient with Joubert syndrome and related disorders. **b** Axial T2-weighted magnetic resonance imaging of the brain showing mildly thickened and elongated superior cerebellar peduncles (arrowheads), a deepened interpeduncular fossa (thin arrow), and vermian hypoplasia (thick arrow)

A 28-year-old Japanese woman who was diagnosed with JSRD and her family members (her parents and her healthy older sister) were involved in this study. The patient was referred to the neurology department for evaluation of her impaired walking and standing at the age of 28 years. She was the second child of non-consanguineous healthy parents (Fig. 1a). She had undergone a living-donor renal transplantation at 8 years of age. Her visual acuity had gradually decreased from infancy, leading to complete blindness. Ophthalmologic examination revealed no light perception in either eye. Neither blepharoptosis nor coloboma was observed. Funduscopic examination showed optic disc pallor. A neurological examination revealed truncal ataxia without apparent limb ataxia. Deep tendon reflexes were normal in all extremities and there were no pathological reflexes. Her Wechsler Adult Intelligence Scale-III verbal IQ score was 75. The clinical symptoms of the patient were summarized in Additional file 1: Table S1. Axial brain magnetic resonance imaging (MRI) showed mildly thickened and elongated superior cerebellar peduncles, a deepened interpeduncular fossa, and vermian hypoplasia, resulting in the molar tooth sign (Fig. 1b). Based on MRI findings and clinical manifestations, the patient was diagnosed with JS with oculorenal defects.

### Molecular genetics

Genomic DNA was extracted from the peripheral blood of each subject using a DNeasy Blood and Tissue Kit (Qiagen, Hilden, Germany). WES of the patient and her family members (her parents and her healthy older sister) was performed using amplicon-based next-generation sequencing. Briefly, libraries were constructed using an Ion AmpliSeq Library Kit v2.0 (Life Technologies, Carlsbad, CA) according to the manufacturer’s instructions. Quantification of the libraries was performed by 2200 TapeStation Instrument using High Sensitivity D1000 Reagents and High Sensitivity D1000 ScreenTape (Agilent Technologies, Santa Clara, CA). Amplified libraries were submitted to emulsion polymerase chain reaction (PCR) using an Ion OneTouch™ 2 Instrument with an Ion PI™ Template OT2 200 Kit v3. Ion sphere particles were enriched using Ion OneTouch ES and were loaded on an Ion PI Chip v2. Sequencing was performed by Ion Proton™ with an Ion PI Sequencing 200 Kit v3. Read sequence files were then run through two independent pipelines constructed with Bowtie2 (http://bowtie-bio.sourceforge.net/bowtie2/index.shtml)-GATK (the Genome Analysis Toolkit; https://www.broadinstitute.org/gatk/index.php) and BWA (Burrows Wheeler Alignment; http://bio-bwa.sourceforge.net/bwa.shtml)-Platypus (http://www.well.ox.ac.uk/platypus) to obtain variant call format files. Then, concordant genetic variants detected by two pipelines were annotated using ANNOVAR program (http://annovar.openbioinformatics.org). Then, variants in 21 known causative genes of JSRD were picked up. To calculate the depth of coverage, we used the GenomicRanges and IRanges packages from Bioconductor (http://www.bioconductor.org), with a sex-considered correction using the average read counts of the X chromosome in each sample, in consideration of the number of X chromosomes. Read counts of the patient were also compared with those of her father as a reference sample in the family. Then, we ran a hidden Markov model algorithm to call CNVs according to the programs described in the eXome Hidden Markov Model (XHMM) [10].

To confirm the mutation identified by WES, Sanger sequencing was performed by amplifying *NPHP1* exon 7 with the following primers: 5′-AATTCCCATCTATGCTCAAGATTT-3′ (forward) and 5′-TTCCCACTTTTGTACCTTTGC-3′ (reverse). PCR products were sequenced using a BigDyeV3.1 terminator Kit on an ABI 310 automated sequencer (Life Technologies).

Digital PCR was performed on a QuantStudio™ 3D Digital PCR System (Life Technologies) according to the manufacturer’s instructions. Briefly, reaction mixtures were prepared with the QuantStudio 3D Digital PCR Master Mix and custom TaqMan probes. Primers for *NPHP1* exon 7 (Hs00138437_cn) and *BUB1* exon 8 (Hs02420021_cn) were purchased from Life Technologies. *BUB1* is located 500 kb upstream of *NPHP1* and was used as a control. Reaction mixtures were loaded onto a QuantStudio 3D Digital PCR Chip, and PCR was performed using recommended conditions. The gene copy number was analyzed using a QuantStudio 3D Digital PCR Instrument.

**Fig. 2:**
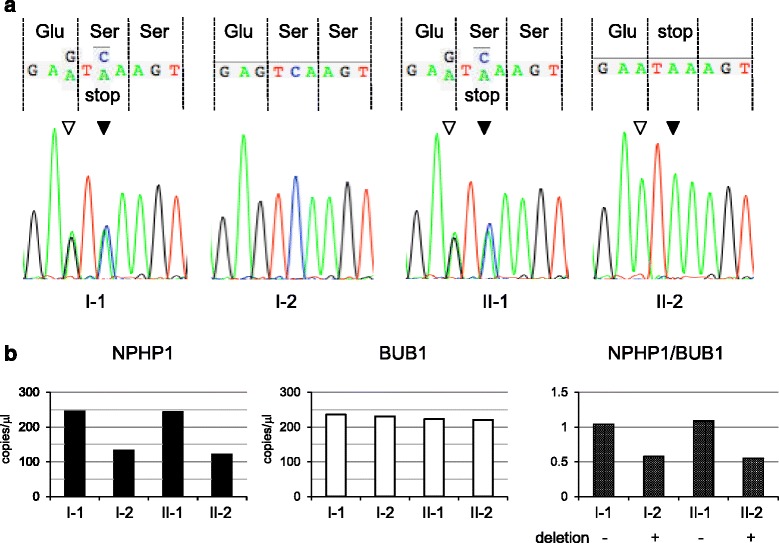
**a** Electropherograms of DNA sequences from family member. Solid arrowhead indicates the c.656C > A mutation. Open arrowhead represents the single nucleotide polymorphism rs11675767. **b** Digital polymerase chain reaction targeted to *NPHP1* and *BUB1*. The number of copies of *NPHP1* (left panel), *BUB1* (middle panel), and the copy number ratios of *NPHP1* to *BUB1* (right panel) are shown

Because JSRD is a genetically heterozygous condition, we performed amplicon-based next-generation sequencing to determine the causative mutations. WES of the patient identified a novel c.656C > A mutation in exon 7 of *NPHP1*, leading to a nonsense mutation at position 219 (p.Ser219*), which was confirmed by Sanger sequencing (NM_000272.3) (Fig. 2a). This mutation was not detected in publicly available databases: Exome Variant Server (ESP6500; http://evs.gs.washington.edu), dbSNP 138 (http://www.ncbi.nlm.nih.gov), 1000 Genomes Profect (http://www.1000genomes.org), ExAC (The Exome Aggregation Consortium; http://exac.broadinstitute.org), and in-house exome sequencing data of 128 control subjects that was obtained using the analysis pipeline described above. Contrary to expectations, we could not detect the c.656C > A mutation in her mother, whereas her father was heterozygous for the mutation. We could not detect any other rare SNVs in the 21 JSRD-related genes in the patient or her family members.

A deletion of *NPHP1* had previously been reported in JSRD [11, 12], so we next performed CNV assessment using a read depth-based CNV detection algorithm. The CNV-calling algorithm predicted the presence of an approximately 470 kb heterozygous deletion including the entire *NPHP1* gene in both the patient and her mother (Additional file 2: Figure S1). To confirm this deletion mutation, digital PCR targeted to *NPHP1* exon 7 and *BUB1* exon 8 was performed. The copy numbers of *NPHP1* (copies/μl) in individuals I-1, I-2, II-1, and II-2 were 245.2, 133.6, 243.7, and 122.6, respectively (Fig. 2b, left panel). This compares with those of *BUB1*: 235.7, 231.0, 223.4, and 221.2, respectively (Fig. 2b, middle panel). The copy number ratios of *NPHP1* to *BUB1* of I-1, I-2, II-1, and II-2 were 1.04, 0.58, 1.09, and 0.55, respectively (Fig. 2b, right panel), indicating the presence of a heterozygous deletion in individuals I-2 (the patient’s mother) and II-2 (the patient). These findings indicated that the patient was compound heterozygous for the paternal nonsense mutation and the maternal deletion mutation.

## Discussion

JSRD is a genetically heterogeneous disorder; therefore, the identification of the causative mutation for each patient by Sanger sequencing is a daunting task even after narrowing down the candidates based on clinical phenotype. As shown here, WES is also useful to detect not only single nucleotide changes but also CNVs that cannot be identified by Sanger sequencing. CNV assessment using WES data of the family led to the detection of a large deletion mutation in combination with a novel nonsense mutation of the remaining allele. In the present case, WES and Sanger sequencing of the patient detected the nonsense mutation in an apparently homozygous state; however, this was refuted by genetic testing to assess CNV. Therefore, it should be noted that analytical pipelines that focus purely on sequence information cannot distinguish homozygosity from hemizygosity because of its inability to detect large deletions. Thus, in this case, comprehensive analysis of the family was necessary to avoid imprecise genetic diagnosis.

CNV detection is an important issue in genetic diagnosis that might be overlooked by conventional sequencing strategies. Although several algorithms capable of identifying CNVs from WES data have been proposed, there is no WES-based CNV-calling method that can detect all CNVs in a large size range [13–15]. Because non-allelic homologous recombination between low-copy repeats on chromosome 2q13 have been shown to be a major cause of large deletions involving the *NPHP1* gene [11, 16, 17], we chose to use XHMM based CNV-calling algorithm, which is suitable for predicting CNVs ranging from 10 kb to 1 Mb [13]. Although we have not determined the precise breakpoint of the deletion, the CNV-calling algorithm was nevertheless successful at predicting the heterozygous large deletion including the entire *NPHP1* gene. It should be noted that WES data could identify not only SNVs but also clinically relevant CNVs without additional experiments such as genome-wide array comparative genomic hybridization or single nucleotide polymorphism array.

CNV validation methods include real-time quantitative PCR and multiplex ligation-dependent probe amplification. In the present case, we performed chip-based digital PCR to confirm the *NPHP1* deletion. This new PCR technique allows absolute quantification of copy numbers without reference to a standard curve, and produces precise results compared with conventional real-time quantitative PCR [18, 19]. We show that digital PCR is an alternative efficient method to confirm clinical relevant CNVs in genetic diagnosis.

## Conclusions

In conclusion, we identified a novel compound heterozygous mutation (p.Ser219*/deletion) in the *NPHP1* gene in a case of JSRD using WES. The present study showed a clinical usefulness of WES and digital PCR to detect clinically relevant CNVs in patients with genetically heterogeneous conditions. It should be noted that patients with genetic disorders might harbor CNVs that cannot be detected by conventional sequencing strategies.

## Additional files

Additional file 1: Table S1. Clinical symptoms of the patient with Joubert syndrome with oculorenal defects in this study. (PPTX 85 kb)

Additional file 2: Figure S1. Schematic presentation of the deletion mutation predicted by a read depth-based copy number variation detection algorithm. The approximately 470 kb heterozygous deletion including the entire *NPHP1* gene is shown in the patient (II-2) and her mother (I-2). The chromosomal positions are based on NCBI build 37. (PDF 84 kb)

## Abbreviations

CNV: Copy number variation
JSRD: Joubert syndrome and related disorders
MRI: Magnetic resonance imaging
OMIM: Online mendelian inheritance in man
PCR: Polymerase chain reaction
SNV: Single nucleotide variant
WES: Whole-exome sequencing
XHMM: eXome hidden markov model
