# Supplementary figures and images for: Whole-exome sequencing and digital PCR identified a novel compound heterozygous mutation in the NPHP1 gene in a case of Joubert syndrome and related disorders
Source: BMC Med Genet. 2017 Mar 27;18:37. doi: 10.1186/s12881-017-0399-2 (PMC5368915; doi:10.1186/s12881-017-0399-2)

## Slide 1
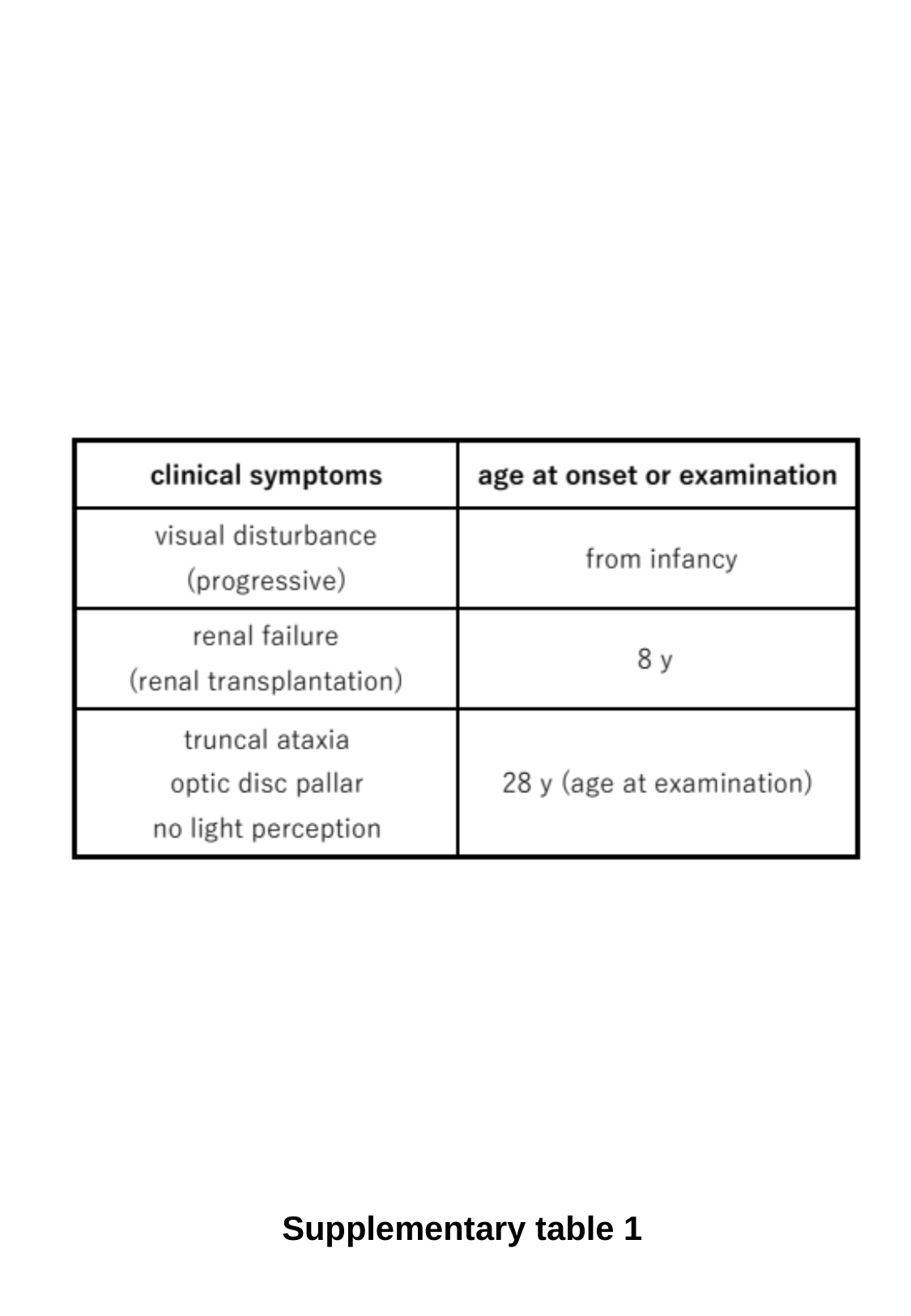

Supplementary table 1

Supplement: Supplementary file 1 — Clinical symptoms of the patient with Joubert syndrome with oculorenal defects in this study. (PPTX 85 kb) [file 12881_2017_399_MOESM1_ESM.pptx]

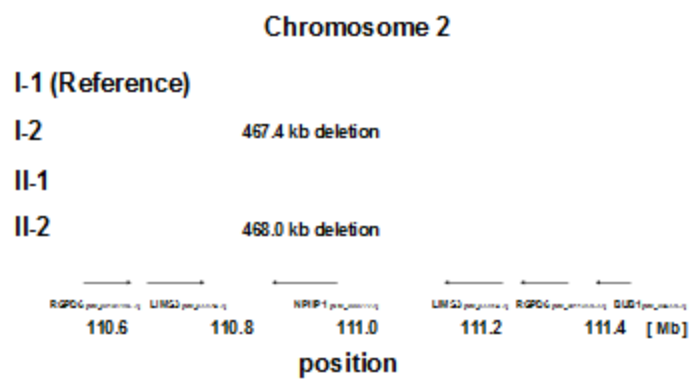

**Supplementary Fig. 1**

Supplement: Supplementary file 2 — Schematic presentation of the deletion mutation predicted by a read depth-based copy number variation detection algorithm. The approximately 470 kb heterozygous deletion including the entire NPHP1 gene is shown in the patient (II-2) and her mother (I-2). The chromosomal positions are based on NCBI build 37. (PDF 84 kb) [file 12881_2017_399_MOESM2_ESM.pdf]
